# Caries induced cytokine network in the odontoblast layer of human teeth

**DOI:** 10.1186/1471-2172-12-9

**Published:** 2011-01-24

**Authors:** Orapin V Horst, Jeremy A Horst, Ram Samudrala, Beverly A Dale

**Affiliations:** 1 Department of Orofacial Sciences, School of Dentistry, University of California, San Francisco, 513 Parnassus Street, San Francisco, CA, 94143, Box 0422, USA; 2 Department of Oral Biology, School of Dentistry, University of Washington, 1959 NE Pacific Street, Seattle, WA, 98195, Box 357132, USA; 3 Department of Endodontics, School of Dentistry, University of Washington, 1959 NE Pacific Street, Seattle, WA, 98195, Box 357448, USA; 4 Department of Microbiology, School of Medicine, University of Washington, 1959 NE Pacific Street, Seattle, WA, 98195, Box 357242, USA

## Abstract

**Background:**

Immunologic responses of the tooth to caries begin with odontoblasts recognizing carious bacteria. Inflammatory propagation eventually leads to tooth pulp necrosis and danger to health. The present study aims to determine cytokine gene expression profiles generated within human teeth in response to dental caries *in vivo *and to build a mechanistic model of these responses and the downstream signaling network.

**Results:**

We demonstrate profound differential up-regulation of inflammatory genes in the odontoblast layer (ODL) in human teeth with caries *in vivo*, while the pulp remains largely unchanged. Interleukins, chemokines, and all tested receptors thereof were differentially up-regulated in ODL of carious teeth, well over one hundred-fold for 35 of 84 genes. By interrogating reconstructed protein interaction networks corresponding to the differentially up-regulated genes, we develop the hypothesis that pro-inflammatory cytokines highly expressed in ODL of carious teeth, IL-1β, IL-1α, and TNF-α, carry the converged inflammatory signal. We show that IL1β amplifies antimicrobial peptide production in odontoblasts *in vitro *100-fold more than lipopolysaccharide, in a manner matching subsequent *in vivo *measurements.

**Conclusions:**

Our data suggest that ODL amplifies bacterial signals dramatically by self-feedback cytokine-chemokine signal-receptor cycling, and signal convergence through IL1R1 and possibly others, to increase defensive capacity including antimicrobial peptide production to protect the tooth and contain the battle against carious bacteria within the dentin.

## Background

Cytokines generate and maintain host responses to microbial infection. Living cells of the host secrete these molecules as paracrine or autocrine signals to recruit cells of the immune system (chemokines), produce inflammation (proinflammatory cytokines), or control the inflammatory responses (anti-inflammatory cytokines). The fine-tuned cytokine networks facilitate the eradication of invading microbes but maintain a balance between pro- and anti-inflammation thereby creating a favorable environment for tissue repair [1].

Dental caries and subsequent tooth pulp inflammation are major oral health problems caused by bacterial infection. Previous studies have reported increased expression of various cytokines in caries-affected dental pulp and/or odontoblasts including transforming growth factor-β1 (TGFβ1), vascular endothelial cell growth factor (VEGF), C-C chemokine ligand 2 (CCL2/MCP1), CCL20/MIP3α, interleukin 8 (IL8/CXCL8), CXC chemokine ligand 10 (CXCL10), epithelial cell-derived neutrophil attractant 78 (ENA78), IL-1β, IL2, IL4, IL6, IL10, IL11, interferon-γ (IFN-γ) and tumor necrotic factor-α (TNF-α) [2-13]. The induction of these cytokines was also shown in cultured pulp-derived fibroblasts and odontoblast-like cells exposed to bacteria or their products *in vitro *[9,10,14-20]. However, these molecular events induced in odontoblast layer (ODL) have not been characterized or distinguished from those of the underlying pulp during the carious process *in vivo*.

Like osteoblasts and other blast cells, the primary function of odontoblasts is generally understood as producing the extracellular structure of dentin. The functional response of odontoblasts to caries is recognized as extending a barrier to the spread of infection by forming reparative dentin. Recently our group and others showed that odontoblasts can also mediate host inflammatory responses to caries directly through production of antimicrobial peptides and cytokines, and indirectly through activation of migratory immune cells using *in vitro *and *ex vivo *models [10,13-15,18-20]. In this study we aim to characterize cytokine expression profiles generated within human teeth in response to dental caries *in vivo*, and to build a mechanistic model of these responses by mapping the *in vivo *differential gene expression of ODL and pulp in healthy (intact) and diseased (carious) teeth across known protein interactions.

We assessed the tooth inflammatory regulatory network as the combined response of cells in ODL and pulp to dental caries *in vivo*. First we described gene expression profiling of cytokines and related immune components in ODL and pulp of normal teeth. Second we analyzed expression changes in carious teeth using cDNA microarrays and verified with quantitative real-time PCR. Additional gene transcripts with significant changes in carious teeth were identified by real-time PCR arrays. We mapped the connectivity between differentially expressed gene transcripts onto a network of experimentally demonstrated protein interactions. The reconstructed interaction network between differentially expressed gene products suggested that ODL amplifies responses through self-feedback cycling of signal-receptor events, building the capacity to dramatically increase cytokine and antimicrobial peptide production to protect the tooth and contain the battle against oral pathogens within the dentin.

## Results

### Gene expression profiling of immune components in the odontoblast layer (ODL) and pulp of normal teeth

Cells in the odontoblast layer (ODL) and pulp of healthy teeth express mRNA of cytokines, chemokines, and their receptors. These expression data were derived from PCR arrays and PCR verification of cDNA arrays. Although many of these genes were detected in both ODL and pulp, multiple genes were preferentially expressed in ODL or the underlying pulp tissue, suggesting good separation of the two components (Table 1). Over 40 of 84 genes were detected in both ODL and pulp of normal teeth.	The most abundantly expressed genes in ODL and pulp of normal teeth were cytokine receptor interleukin 1 receptor 1 (IL1R1), C-X-C family chemokine ligand 12 (CXCL12), CXCL14, and macrophage migration inhibitory factor (MIF). These genes were detected by real-time quantitative PCR (qPCR) before 25 amplification cycles, which indicates more abundant expression than other genes detected at the greater amplification cycles.

**Table 1:** Gene expression profile of cytokines and their receptors in the odontoblast layer (ODL) and pulp of normal teeth*.

ODL > PULP	PULP > ODL
BCL6, CEBPB, ICEBERG, IL1R1, IL5RA, IL8, MIF, SPP1, TOLLIP	C3, CCL16, CCL19, CCL2, CCL21, CCL23, CCL25, CCL4, CCL5, CCL8, CCR2, CCR3, CCR5, CX3CR1, CXCL1, CXCL12, CXCL14, CXCL2, CXCL5, IL5, IL9, LTB4R, TNFA, CD40LG, XCR1

Abbreviations: BCL6, B-cell CLL/lymphoma 6; CEBPB, Enhancer binding protein beta; ICEBERG, ICEBERG caspase-1 inhibitor; IL1R1, Interleukin 1 receptor, type I; IL5RA, interleukin 5 receptor alpha; IL8, Interleukin 8; MIF, Macrophage migration inhibitory factor; SPP1, Secreted phosphoprotein 1 (osteopontin, bone sialoprotein I); TOLLIP, Toll-interacting protein; C3, complement component 3; CCL16, Chemokine (C-C motif) ligand 16; CCR2, Chemokine (C-C motif) receptor 2; CX3CR1, Chemokine (C-X3-C motif) receptor 1; CXCL1, Chemokine (C-X-C motif) ligand 1; LTB4R, Leukotriene B4 receptor; TNFA, Tumor necrosis factor alpha; CD40LG, CD40 ligand; XCR1, Chemokine (C motif) receptor 1; XCR1, chemokine (C motif) receptor 1.

*These data were obtained from PCR arrays and qPCR verification of cDNA array data including CCR2, CCR4, CCR5, CCR9, CCL3, CCL23, IL1B, TNFA, LTA, IL1R1, MIF, CXCL12, and CXCL14.

In normal teeth, mRNA expression of most genes was higher in the pulp, although expression was higher in ODL for the following genes (Table 1): IL1R1, MIF, toll-interacting protein (TOLLIP), transcription factor 5 or CCAAT/enhancer binding protein beta (CEBPβ/CEBPB), B-cell lymphoma 6 (BCL6), iceberg caspase-1 inhibitor (ICEBERG), IL5Rα/IL5RA, IL8, and osteopentin (OPN/SPP1). C-C family chemokine receptor 3 (CCR3) mRNA was detected in the pulp but not in ODL.

### Caries-induced inflammatory gene expression in ODL and underlying pulp

**Figure 1:**
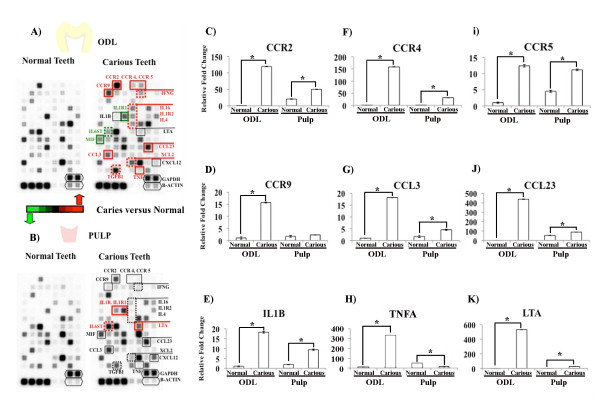
**cDNA array analysis for cytokines, chemokines, and receptors involved in the responses of teeth to caries**. Expression of inflammatory genes in the odontoblast layer (ODL) and pulp of normal versus carious teeth was determined by cDNA arrays (A&B) and verified by real-time quantitative PCR (qPCR) (C-K). C-C chemokine receptor 2 (CCR2), CCR4, CCR5, C-C chemokine ligand 3 (CCL3), CCL23, interleukin-1β (IL-1B), tumor necrosis factor α (TNFA), and lymphotoxin α (LTA) mRNA significantly increased in ODL and pulp of carious teeth. CCR9 was significantly upregulated only in the ODL of carious teeth. These genes may play important roles in the responses of teeth to caries. Black Hexagons indicate house keeping genes (glyceraldehyde 3-phosphate dehydrogense, GAPDH; and beta-actin, B-ACTIN). Black-boxed marks indicate no statistically significant changes in gene expression levels between normal and carious teeth. Red-boxed marks label genes with statistically significant increases in carious teeth while green-boxed marks label genes with statistically significant decreases in carious teeth. Genes labeled with a solid line box were verified by qPCR but those with a dashed line box were not verified by qPCR. Values are reported as relative fold change in mRNA transcription of normal versus carious samples. The data represent means and standard errors from triplicate wells of one experiment and are representative of three independent experiments. Asterisks indicate statistically significant changes, with P < 0.05.

**Figure 2:**
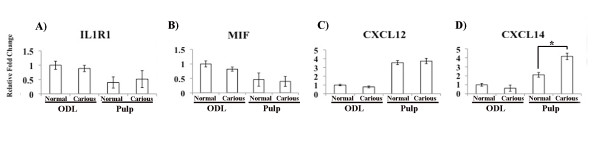
**The effect of caries on the transcription of genes highly expressed in normal teeth**. Expression of IL1R1 (A), MIF (B), CXCL12 (C), and CXCL14 (D) in ODL and pulp of normal versus carious teeth was determined by real-time PCR. Values are reported as relative fold change in mRNA transcription of normal versus carious samples. The data represent means and standard errors from triplicate wells of one experiment and are representative of three independent experiments. Asterisks indicate statistically significant changes, with P < 0.05.

**Figure 3:**
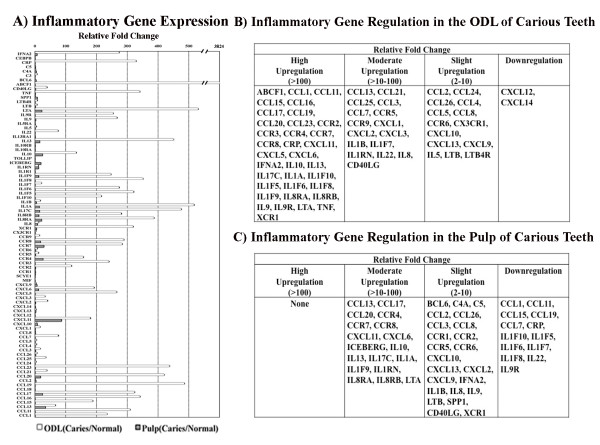
**Quantitative analysis for differential changes in the odontoblast layer (ODL) and pulp of carious teeth**. Expression of inflammatory genes in ODL and pulp of normal versus carious teeth was determined by real-time quantitative PCR arrays (A-C). The fold change of gene expression detected by PCR arrays was more robust than that determined by cDNA arrays. However, the overall regulatory profile was similar: the increase of inflammatory gene expression in carious teeth was much more profound in ODL than in the pulp. Values are reported as relative fold change in mRNA transcription of carious versus normal samples. The data represent means and standard errors from triplicate sets of array analyses. Abbreviations: IFNA2, Interferon alpha 2; CEBPB, Enhancer binding protein beta; CRP, C-reactive protein; C4A, complement component 4A; BCL6, B-cell CLL/lymphoma 6; ABCF1, ATP-binding cassette, subfamily F member 1; CD40LG, CD40 ligand; TNF, Tumor necrosis factor alpha; SPP1, Secreted phosphoprotein 1 (osteopontin, bone sialoprotein I, early T-lymphocyte activation 1); LTB4R, Leukotriene B4 receptor; LTB, Lymphotoxin beta; LTA, Lymphotoxin alpha; TOLLIP, Toll-interacting protein; ICEBERG, ICEBERG caspase-1 inhibitor; IL, Interleukin; IL1R1, Interleukin 1 receptor, type I; IL1RN, Interleukin 1 receptor antagonist; IL10RA, interleukin 10 receptor alpha; IL10RB; interleukin 10 receptor beta; IL1F9, Interleukin 1 family member 9; XCR1, Chemokine (C motif) receptor 1; CX3CR1, Chemokine (C-X3-C motif) receptor 1; CCR, Chemokine (C-C motif) receptor; SCYE1, Small inducible cytokine subfamily E member1 (endothelial monocyte activating); MIF, Macrophage migration inhibitory factor; CXCL, Chemokine (C-X-C motif) ligand; CCL, Chemokine (C-C motif) ligand.

A profound increase in expression of inflammatory genes in carious teeth examined here occurred in ODL mainly, while fewer differences were found for the pulp, as shown by cDNA arrays (Figure 1) and real-time PCR (Figures 1, 2, 3). cDNA arrays showed increased expression of 13 genes in ODL while 4 genes were up-regulated in the pulp (Figures 1A &1B). Up-regulation of CCR2, CCR4, CCR5, CCR9, CCL3, CCL23, and TNFA in ODL of carious teeth was confirmed by qPCR (Figures 1C, D, F, G, H, I, and 1J respectively). While cDNA arrays failed to detect any changes of these genes in the pulp of carious teeth, qPCR revealed significant increases of CCR2, CCR4, CCL3, CCR5, and CCL23 (Figures 1C, F, G, I, and 1J respectively). Similarly, qPCR detected significant increases of IL-1β/IL1B, TNF-α/TNFA, and LTA in both ODL and pulp of carious teeth but cDNA arrays only revealed significant increases of IL-1β/IL1B and LTA in the pulp and TNF-α/TNFA in ODL of carious teeth (Figures 1A, B, E, H, and 1K respectively). The qPCR verification data are consistent with those from PCR arrays.

As mentioned above, IL1R1, MIF, CXCL12, and CXCL14 presented the most abundant expression in ODL and pulp of normal teeth. In carious teeth, MIF and IL1R1 decreased slightly in ODL as shown by cDNA arrays (Figure 1) and qPCR (Figure 2). However, these changes were not significant. The expression of CXCL12 was not significantly altered in ODL and pulp of carious teeth. Only CXCL14 significantly increased in the pulp but not ODL of carious teeth (Figure 2).

Among chemokines, pro-inflammatory, and anti-inflammatory mediators as well as their receptors examined in this study, the ATP-binding cassette subfamily F member 1 (ABCF1) was the most highly up-regulated gene in ODL of carious teeth. This gene was not detected in either ODL or the pulp of normal teeth. ABCF1 expression did not change in the pulp of carious teeth (Figures 3B and 3C). Other inflammatory mediators differentially regulated in ODL and pulp of carious teeth are shown in Figure 3.

### Protective production of antimicrobial peptides induced by pro-inflammatory mediators increased in ODL of carious teeth

We examined the effects of IL-1β/IL1B, TNF-α/TNFA, IFNγ/IFNG, and TLR4 activation on antimicrobial peptide production using *in vitro *cultured human odontoblast-like cells. The protein products of these genes are major inducers of pulpal inflammation and are well known to regulate production of other cytokines.

**Figure 4:**
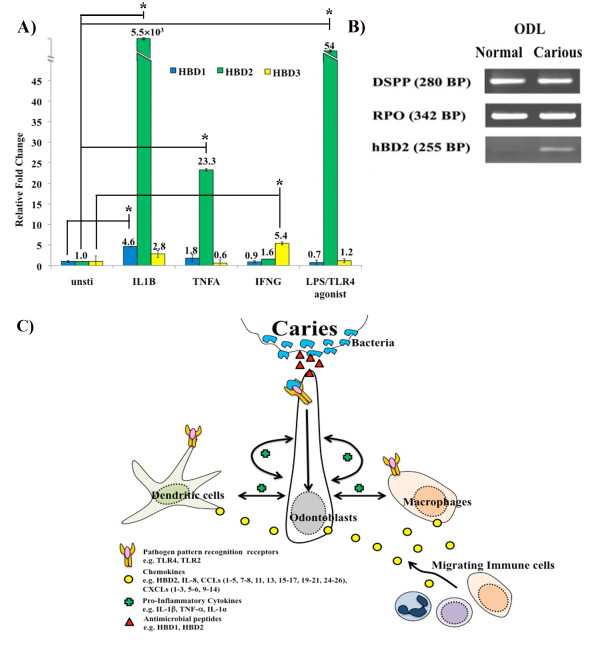
**Innate immunity in the odontoblast layer (ODL)**. The effect of IL-1β/IL1B, TNF-α/TNFA, IFNγ/IFNG, and LPS on antimicrobial peptide regulation determined by real-time quantitative PCR (A). The effect of caries on HBD2 mRNA expression was detected by semi-quantitative PCR (B). IL-1β, TNF-α, and LPS amplify HBD2 transcription in odontoblast-like cells *in vitro*. Consistently, caries increase expression of HBD2 mRNA in cells of ODL *in vivo*. Values are reported as relative fold change in mRNA transcription of unstimulated versus stimulated samples. The data represent means and standard errors from triplicate wells of one experiment and are representative of at least three independent experiments. Asterisks indicate statistically significant changes, with P < 0.05. A diagram illustrates the proposed caries-induced cellular interactions among cells in ODL through pro-inflammatory cytokines and chemokines (C). Bacterial components from caries activate cytokine/chemokine release from odontoblasts, dendritic cells, and/or macrophages via toll-like receptors (TLRs). Proinflammatory cytokines released from these cells act as autocrine and paracrine signals to amplify cytokine responses including antimicrobial peptide, cytokine, and chemokine production. The release of chemokines creates a migration gradient for immune cells to ODL while antimicrobial peptides reduce bacterial load.

Pro-inflammatory cytokines IL-1β and TNF-α but not IFNγ up-regulated mRNA transcription of human β-defensin 2 (HBD2) in a similar manner to TLR4 activation (Figure 4A). The magnitude change of HBD2 up-regulation by IL-1β (5.5×10^3^-fold increase) was much more robust than those induced by TNF-α (23.3-fold increase) and bacterial LPS (54-fold increase). HBD2 transcription was also increased in ODL of carious teeth (Figure 4B). HBD1 gene transcription slightly increased in the presence of IL-1β (Figure 4A) and in ODL of carious teeth (data not shown). HBD3 mRNA slightly increased in the presence of IFNγ (Figure 4A). However, the gene transcription of HBD3 was not significantly changed in ODL and pulp of carious teeth (data not shown).

### Model of caries signal induction

The gene expression analysis findings suggest cell-cell interaction events mediated by pro-inflammatory cytokines, chemokines, and antimicrobial peptides that are predicted to build the immune defense capacity within ODL to protect the tooth and contain the battle against oral pathogens in dentin (Figure 4C).

### Mapping of caries-induced molecular interactions for ODL inflammatory responses

**Figure 5:**
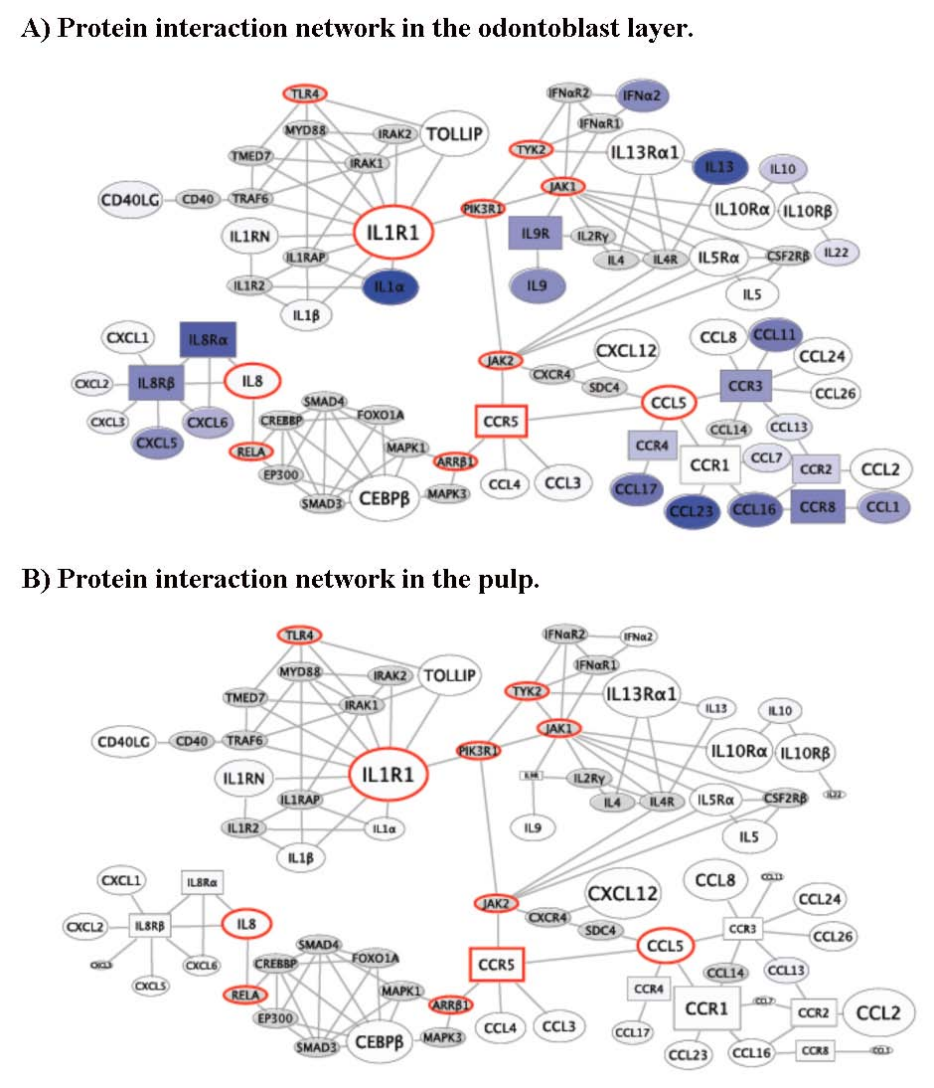
**The minimal connectivity of genes increased in the odontoblast layer (ODL) and pulp of carious teeth**. This network shows known direct or one off interactions between genes measured to be significantly upregulated in this study. For each gene, the expression level (inverse of PCR threshold cycle) in ODL (A) and pulp (B) of carious teeth is rendered as node size. Differential expression in ODL (A) and pulp (B) of carious teeth versus normal teeth (fold increase) is depicted as a color heat map with white showing no change and saturated blue meaning greater up-regulation in carious ODL (A) and pulp (B). Network bottlenecks are highlighted in red, signifying the most important candidate inflammatory signal mediators: PIK3R1, IL1R1, TLR4, ARRβ1, CCL5, CCR5, IL8, JAK1, JAK2, RELA, and TYK2. The key receptors for inflammatory signals induced by caries in ODL appear to converge through IL1R1, CCR5, and IL8Rα/β. The gene expression data used for building this map were derived from PCR arrays and qPCR verification data of cDNA arrays.

**Figure 6:**
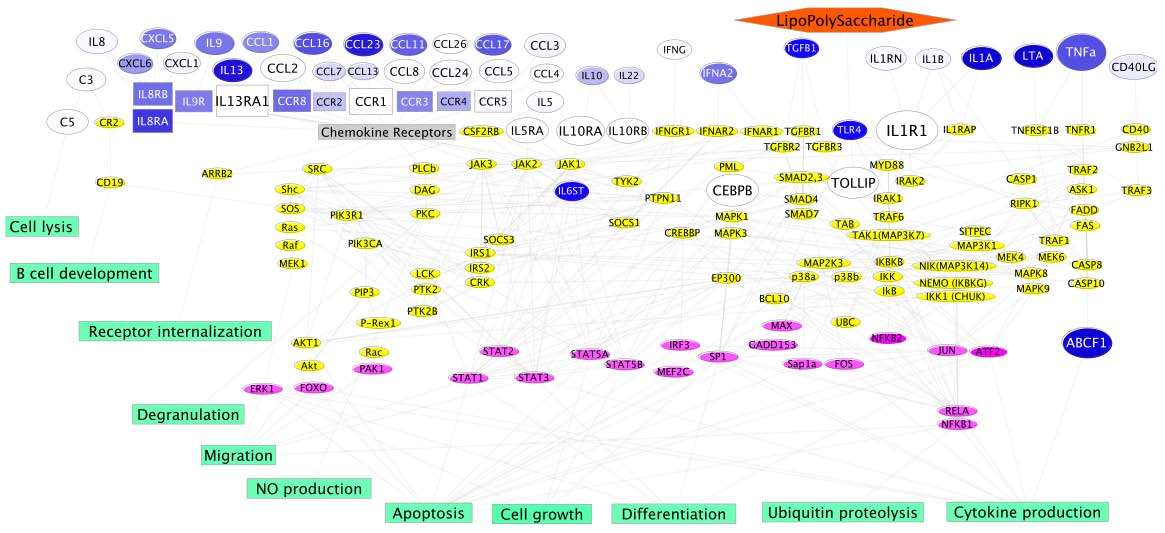
**A model of molecular interactions in the odontoblast layer (ODL) of carious teeth**. We map the inflammatory signal arising from ODL in response to caries as the network of previously demonstrated protein interactions, downstream from the differentially regulated gene products observed in this study. These activating or inhibitory interactions taken from the STRING and KEGG databases have been demonstrated for the same human proteins in other tissue and cell types. Size and color of node depiction for caries-induced genes in ODL were varied as in Figure 4. Intracellular signaling molecules (yellow) were mapped and linked to the transcription factors (magenta) and the resulting cellular responses (green). The network describes a parts list of component interactions to be assessed in future studies for the mechanistic basis of signal propagation from caries to pulpitis.

A model of the downstream molecular mechanisms and resulting cellular responses to dental caries in ODL was built by integrating the differential gene expression profile with previously defined functional interactions and signaling pathways (Figures 5 and 6).

A few receptors receive the signal of abundant ligands, and are themselves abundant in ODL of carious teeth including IL1R1, IL8Rα, IL8Rβ, IL9R, IL13Rα1, CCR1, CCR2, CCR3, CCR4, and CCR8. The dramatic increase of either receptors or ligands in ODL (Figure 5A) but not in the pulp (Figure 5B) of carious teeth suggests: 1) signals through these receptors are critical to initiate inflammatory events within ODL, and 2) the propagation of these signals into the pulp may lead to the development of irreversible pulpitis in carious teeth.

The global downstream signaling network from bacterial signals and host cytokines, *via *their receptors and signaling molecules, to the transcription factor activation and consequence cellular responses is shown in Figure 6. Some of these responses are features related to the classic pulp pathologic progression from infection (caries) to inflammation and to necrosis, including cytokine production, inflammatory cell migration, nitric oxide production, proteolysis, cell lysis, and apoptosis. Other responses include the convergence of many pathways onto PIK3R1 (85 kD regulatory subunit of phosphatidylinositol 3-kinase) and PIK3CA (110 kD catalytic subunit of phosphatidylinositol 3-kinase), suggesting that modulation of phosphatidylinositol phosphorylation by these proteins could present a mechanism to control the inflammatory responses.

## Discussion

Odontoblasts form the peripheral layer of the dental pulp as an internal host-microbial interface for the tooth, and thus are vulnerable and need the capability of innate host defenses [13,18]. Cytokines and chemokines mediate the crosstalk between odontoblasts and cells of the innate immune system such as neutrophils, monocytes/macrophages, dendritic cells, and natural killer cells. These proteins are secreted by both odontoblasts and immune cells in response to bacterial stimuli to attract additional immune cells as well as initiate and modulate inflammatory responses. Here we propose a mechanistic model of cytokine-signaling network in the odontoblast layer (ODL) of human teeth in response to dental caries and the role of IL1R1 and ligands IL-1β and IL-1α in carrying the converged inflammatory signals to amplify innate immune responses including the production of antimicrobial peptides to protect the tooth and contain the battle against carious bacteria within dentin. We also show that cells in ODL of normal and carious teeth expressed mRNA for various immune components of which the majority measured here are chemotactic cytokines. In response to carious infection, these cytokines are highly up-regulated in ODL and most likely induce leukocyte migration into the tooth to increase immunologic capacity. This finding is supported by previous data *in vitro *that protein secretions from odontoblast-like cells exposed to bacterial products induced migration of monocyte-derived immature dendritic cells [10,15].

Our findings of active immune components in healthy teeth expand upon previous findings. One study using healthy teeth reported mRNA expression of TGFα/TGFA, CCR2, CXCL1, and CXCL6 only in ODL, and CCL5, CCL15, and LTB gene expression only in the pulp [21]. Conversely, in this study we found expression of all these markers in both ODL and underlying pulp of normal teeth. Other studies reported mRNA expression of CCL2, CCL26, CXCL12, CXCL14, IL8Rβ/IL8RB, LTB4R, and SCYE1 in cultured human odontoblast-like cells [10,15], which matches our *in vivo *results from ODL of normal teeth.

Odontoblasts recognize carious bacteria and initiate immune responses through toll-like receptors (TLRs) [10,13-15,18]. TGFβ1 was shown to attenuate odontoblast inflammatory responses by inhibiting TLR2 and TLR4 expression, which maintain homeostasis within the tooth during carious infection [18]. We also noticed other TLR signal antagonists in the tooth including Toll-interacting protein (TOLLIP) and IL10 [22,23]. High expression of TOLLIP in ODL can provide a negative feedback loop for TLR-mediated inflammation to protect the underlying pulp. IL10 and receptors, IL10Rα/IL10RA and IL10Rβ/IL10RB were present in ODL and pulp. IL10 was highly up-regulated in ODL of carious teeth (>100 fold) and provides another mechanism to attenuate pulp inflammatory responses.

Our hypothesis that ODL is the primary biologic unit of immune responses in the tooth is supported by the profound increase in expression of many inflammatory genes within ODL but not in the pulp. As we do not assess any of the cell types alone, this immune modulatory tissue includes odontoblasts and immune cells such as dendritic cells, macrophages, lymphocytes, and neutrophils. These responses are mediated by cell-to-cell interactions within ODL, and imply differences between *in vitro *and *in vivo *responses to carious bacteria. Our findings together suggest that ODL is effective in attenuating carious infections thereby limiting the inflammatory changes within ODL and maintaining the pulp in a relatively protected environment.

In the presence of bacteria, odontoblasts secrete various chemotactic cytokines for neutrophils, monocytes/macrophages, immature dendritic cells, and lymphocytes including interleukin 8 (IL-8), chemokine (C-C motif) ligand 2 (CCL2), CCL7, chemokine (C-X-C motif) ligand 2 (CXCL2), and CXCL10 [10,13,24,25]. Similarly we found up-regulation of these genes in ODL of carious teeth. CXCL2 and CXCL10 mRNA also increased in the pulp tissues of carious teeth but CCL7 slightly decreased.

Other chemokines increased in ODL of carious teeth are CCL1, CCL3-5, CCL8, CCL11, CCL13, CCL15-17, CCL19-21, CCL23-25, CXCL1, CXCL3, CXCL5, CXCL6, CXCL9-11, and CXCL13. The resulting gradient of these chemokines attracts more migration of immune cells into the tooth [26]. The migratory immune cells, in particular monocytes/macrophages, release a large amount of pro-inflammatory cytokines such as IL-1β, TNF-α, IL-6, and IL-12, which regulate inflammatory reactions in the tissue [27]. We previously showed that human odontoblasts increased transcription of pro-inflammatory cytokines, IL-1β and TNF-α in response to bacterial infection *in vitro *[13]. Here we show that these pro-inflammatory cytokines and others including CRP, ABCF1, IL9, LTA, LTB, IL1A, IL17C, IL1F10, and IL13, were also increased in ODL of carious teeth *in vivo*.

We attempted to identify candidate signal propagators by mapping caries-induced expression of inflammatory mediators onto an experimentally verified set of protein interactions. Network analysis shows IL1R1 standing out as a possible early amplifier of the caries signal, as one of the most abundantly expressed genes in ODL with or without caries induction (Figure 5A). The well known pro-inflammatory and immunoregulatory cytokine IL1R1 agonists, IL-1α and IL-1β, are both highly expressed by cells in carious ODL. IL-1α is the third most up-regulated gene after ABCF1 and LTA (Figure 5A). The signal propagation from IL1R1 overlaps with the TLR4-activated NFkB pathway, suggesting direct signal amplification (Figure 6).

We demonstrate that activation of IL1R1 by IL-1β (or IL-1α) may carry an important activation signal for innate immune responses, with the example of antimicrobial peptide production (Figure 4). The important role of IL1R1 in protecting the tooth and surrounding bone from polymicrobial infection (*Streptococcus mutans*, *Streptococcus intermedius*, *Peptostreptococcus micros*, *Porphyromonas gingivalis*, *Prevotella intermedia*, and *Fusobacterium nucleatum*) was verified *in vivo *by using genetically modified IL1R1 knockout mice. Pulp tissues of teeth experimentally infected with mixed bacteria became necrotic faster and had greater bacterial invasion in IL1R1-null mice than wild-type controls. Further abscess formation and the loss of surrounding bone around infected teeth were shown to be greater in IL1R1-null mice than wild-type controls [28,29].

Although cDNA arrays showed a reduction of IL1R1 in ODL of carious teeth, qPCR data indicated that this change was very low and not statistically significant. We also observed similar result for TLR4 expression in ODL of carious teeth (unpublished observation). TLR4 activation amplifies inflammatory signaling through the activation and production of NF-κB, avoidance of ATF3, and cyclic activation of C/EBPδ [30]. While the levels of C/EBPδ, ATF3, and NF-kB control the IL6 output, the supra-threshold level of TLR4 does not affect signal amplification. Moreover, flux in TLR4 production would confuse C/EBPδ interpretation of a transient signal as a persistent signal. We present IL1R1 as playing a similar role in inflammatory signal amplification to that of TLR4.

ABCF1, the most highly upregulated gene in ODL of carious teeth was mapped downstream of TNF-α and caspase 10. This gene is regulated by TNF-α [31] and cleaved by caspase 10 [32]. Little is known about functions of this gene but it was shown to regulate protein synthesis [33], inflammatory progression [34], and apoptosis [32]. Activation of initiator caspases including caspase 8 and 10 during apoptosis could result in the cleavage of ABCF1 and subsequent regulation of apoptotic signaling. The dramatic up-regulation of ABCF1 in ODL of carious teeth may prime the surrounding cells of the ODL for necrosis.

The signaling pathways from TLR4, TGFβ, chemokine, interleukin, and TNF receptors were mediated through various signaling molecules such as MYD88, IKK, TRAF, Smad, MAP kinase, JAK/STAT, and caspases with high interactions and cross-talk among these signaling pathways. Output from this network includes various aggregate cellular responses including the convergence of many pathways onto PIK3R1 (85 kD regulatory subunit of phosphatidylinositol 3-kinase) and PIK3CA (110 kD catalytic subunit of phosphatidylinositol 3-kinase), suggesting that modulation of phosphatidylinositol-3 kinase (PI3K) activity by these proteins could present a mechanism to control the inflammatory responses. Consistent with this hypothesis, inhibition of PI3K in odontoblast-like cells exposed to carious bacteria (i.e *Streptococcus mutans*) significantly reduced the transcription of inflammatory cytokines IL6 and IL8 [35].

## Conclusions

Cells in the odontoblast layer initiate immunologic responses of the tooth to dental caries through proinflammatory cytokine and chemokine signaling. The model we propose for this cytokine interaction network suggests multiple candidate mediators of signal propagation to irreversible inflammatory damage. The cytokine signaling network reported here provides a map to guide future studies to identify diagnostic or therapeutic targets for pulpal inflammation.

## Methods

### Sample Collection and Preparation

Thirty-two freshly extracted human third molars with complete root formation were collected from patients with consent following an approved protocol of the University of Washington Human Subjects Review Board. Sixteen teeth were intact while the other 16 teeth had moderate to deep caries involving 1/2 to 2/3 of the dentin thickness. The isolation of odontoblast layer (ODL) and underlying pulp was performed as previously described [13,36,37].

The periodontal tissues were mechanically removed under a dissecting microscope. The tooth was rinsed with 5.25% NaOCl to ensure complete removal of periodontal tissues. Then the tooth was rinsed with DNase RNase free water and was submerged in phosphate buffered saline (PBS) while a horizontal groove was made 1-2 mm above the roots. The roots were then split off and the loose core of pulp tissue was pulled out, leaving ODL attached to the tooth crown. Both the pulp tissue and crown ODL were placed in RNA Later (Ambion, Austin, Texas) until processed for RNA isolation.

After all pulp and ODL tissues were removed for RNA isolation, the non-decalcified teeth were sectioned into two halves for direct examination of carious infection depth. A spoon excavator was used to remove caries until hard dentin was reached. The depth from the dentin enamel junction to the pulp (dentin thickness) was evaluated in sixths. Teeth with excavation reaching 1/2 to 2/3 of the dentin thickness were selected for use in this study.

Three pooled samples (one from three teeth, another from three teeth, and another from four teeth) were prepared for cDNA array analysis for each tissue group (normal ODL, normal pulp, carious ODL, and carious pulp). Each remaining pooled RNA preparation was analyzed in qPCR verification experiments. Three pooled samples of two teeth each were prepared for the qPCR array for each tissue group. In total, 10 normal teeth (pulp and ODL), and 10 carious teeth (pulp and ODL) were analyzed by cDNA and qPCR verification, and 6 normal teeth (pulp and ODL), and 6 carious teeth (pulp and ODL) were analyzed by PCR arrays.

### Cell Culture

An *in vitro *model of human odontoblasts (hOD) was created and characterized in our previous study [18]. Briefly, cells were maintained at 37°C in a 5% CO2 atmosphere in Dulbecco's Modified Eagle's Medium (DMEM)/Nutrient Mixture F-12 without HEPES (Gibco, Grand Island, NY, USA), supplemented with 10% heat-inactivated fetal bovine serum (FBS, Hyclone, Logan, UT, USA), 1 μg/mL of vitamin K1 (Sigma-Aldrich, St. Louis, MO, USA), 50 μg/mL of ascorbic acid (Sigma-Aldrich), 100 I.U./mL of penicillin G, 100 μg/mL of streptomycin sulfate (Gibco), 0.3 μg/mL of fungizone (Gibco), 1% 100X insulin-transferrin-selenium-X (Gibco), and 10 mM β-glycerophosphate (Sigma-Aldrich). A library of *in vitro *odontoblast-like cell clones was established. Each clone was grown from a single cell. From previously described clones [18], the hOD2 clone was chosen because expression of odontoblast markers DSPP and DMP1 is relatively similar to native human odontoblasts, and higher than other clones we evaluated. The hOD2 cells were stimulated with each of the three pro-inflammatory cytokines IL-1β (100 ng/ml, Catalog# PHC0814, BioSource, Invitrogen, Carlsbad, CA), TNF-α (100 ng/ml, Catalog# CRT100A, Cell Sciences, Canton, MA), IFNγ (100 ng/ml, Catalog# CRI000A, Cell Sciences) or TLR4 agonist (100 ng/ml, Purified E. coli LPS). Specificity of TLR4 activation by *E. coli *LPS used in this study was verified in our previous study [13].

### RNA Isolation and cDNA Microarray

Total RNA of ODL and pulp was separately extracted by using Trizol^® ^Reagent (Invitrogen, Carlsbad, CA), treated with RNase-free DNase (Qiagen, Valencia, CA), and purified by using RNeasy^® ^minikit (Qiagen).

Total RNA was isolated from cultured cells and purified using similar methods. To verify adequate RNA quality, RNA integrity numbers of all samples were confirmed to be more than 7.0 by using Agilent 2100 Bioanalyzer (Agilent Technologies, Palo Alto, CA). The cDNA arrays (GEArray™ Q series human inflammatory cytokines and receptors HS-015) were processed and analyzed according to the manufacturer's instruction (SA Biosciences, Frederick, MD) and as previously described [10,38,39].

Briefly, each cDNA probe was synthesized from 1.5 μg of total RNA using RT-Labeling kit (SA Biosciences, Frederick, MD). Then the cDNA probe was labeled with biotin using the AmpoLabeling-LPR kit (SA Biosciences) according to the manufacturer's instruction. The cDNA probe was hybridized onto each array membrane (GEArray™ Q series human inflammatory cytokines and receptors HS015, SA Biosciences). The chemiluminescent signals on each array were detected by exposing the array membrane onto an X-ray film, which was processed, scanned, and saved into TIFF image file. The chemiluminescent signals were quantified and analyzed by using the SA Bioscience microarray software analysis suite. The analyses were performed on a triplicate set of cDNA arrays using three different pooled samples of normal ODL, normal pulp, carious ODL, and carious pulp.

The genes for cytokines and receptors included in the cDNA arrays are BLR1, CCR1, CCR2, CCR3, CCR4, CCR5, CCR6, CCR7, CCR8, CCR9, XCR1, CX3CR1, CXCR4, IFNG, IL10, IL10RA, IL10RB, IL11, IL11RA, IL12A, IL12B, IL12RB1, IL12RB2, IL13, IL13RA1, IL13RA2, IL15, IL15RA, IL16, IL17A, IL17RA, IL18, IL18R1, IL1A, IL1B, IL1R1, IL1R2, IL2, IL20, IL21, IL25, IL2RA, IL2RB, IL2RG, IL4, IL5, IL5RA, IL6, IL6R, IL6ST, IL9, IL9R, LEP, LTA, LTB, LTBR, MIF, CCL1, CCL11, CCL13, IL3, CCL15, CCL16, CCL17, CCL18, CCL19, CCL2, CCL20, CCL21, CCL22, CCL23, CCL24, CCL25, CCL3, CCL4, CCL5, CCL7, CCL8, CXCL10, CXCL11, CXCL13, CXCL5, CXCL6, XCL1, XCL2, CX3CL1, SCYE1, CXCL12, SDF2, TGFA, TGFB1, TGFB2, TGFB3, TNFA, TNFRSF1A, TNFRSF1B.

### Quantitative Real-Time Polymerase Chain Reaction (qPCR)

PCR reagents and all PCR primers for cytokines and receptors except those for detecting β-defensin genes were purchased from SA Biosciences. Specific primers for human β-defensin 1 (HBD1), HBD2, HBD3, and house keeping genes GAPDH are as follows: HBD1 sense, 5"- CAC TTG GCC TTC CCT CTG TA-3"; HBD1 antisense, 5"-CGC CAT GAG AAC TTC CTA CC-3"; HBD2 sense, 5'-CCA GCC ATC AGC CAT GAG GGT-3'; HBD2 antisense, 5'-GGA GCC CTT TCT GAA TCC GCA-3"; HBD3 sense, 5"-GTG AAG CCT AGC AGC TAT GAG GAT-3"; HBD3 antisense, 5"- TGA TTC CTC CAT GAC CTG GAA-3"; GAPDH sense, 5"-CAA AGT TGT CAT GGA TGA CC -3"; and GAPDH antisense, 5"-CCA TGG AGA AGG CTG GGG-3".

The quantitative real-time PCR (qPCR) amplification was performed using the iCycler system (Bio-Rad, Hercules, CA). Briefly, 1 μg of total RNA was used for cDNA synthesis using RT^2 ^First Strand kit (SA Biosciences). 1 μl of the resulting cDNA product was used for each PCR reaction.

Each PCR reaction contains 12.5 μl of RT^2 ^SYBR Green qPCR Master Mixes (PA-011, SA Biosciences), 10.5 μl of nuclease-free water, 1 μl of cDNA, and 1 μl of 10 μM PCR primers. Thermocycling conditions were initial denaturation at 95°C for 10 min and amplification at 40 cycles of 95°C for 15 sec, followed by 60°C for 1 min. PCR analyses were performed in triplicates and repeated three times using three different pooled samples. GAPDH was used as house keeping gene. In initial experiments, amplification efficiency was determined for all primer pairs. Melt curve analysis confirmed a single specific PCR product from each primer pair. The threshold cycle number was determined on the amplification plot during the early log phase of product accumulation at which the fluorescence clearly rises above background in a straigth line. Quantification was performed using the comparative Ct method as previously described [40], using the amplification efficiency determined for each primer pair, and that compared to GAPDH.

For PCR arrays (PAHS-011: Human Inflammatory Cytokines & Receptors PCR Array, SA Biosciences), 91 μl of nuclease free water was added into 20 μl of the resulting cDNA product to make 111 μl volume. Then 102 μl of the cDNA was used for 96 PCR reactions containing 1150 μl of RT^2 ^SYBR Green qPCR Master Mixes (PA-011, SA Biosciences), and 1048 μl of nuclease-free water. 20 μl of this mixture was loaded into each well of a PCR array plate containing each specific PCR primer pair. Thermocycling conditions were initial denaturation at 95°C for 10 min and amplification at 40 cycles of 95°C for 15 sec, followed by 60°C for 1 min. PCR array analyses were performed in triplicates using three different pools of each sample type. Relative fold changes of gene expression were calculated using the Delta-Delta Ct quantification method (Web-Based PCR Array Data Analysis software, SA Biosciences).

The genes for cytokines and receptors included in the PCR arrays are ABCF1, BCL6, C3, C4A, C5, CCL1, CCL11, CCL13, CCL15, CCL16, CCL17, CCL18, CCL19, CCL2, CCL20, CCL21, CCL23, CCL24, CCL25, CCL26, CCL3, CCL4, CCL5, CCL7, CCL8, CCR1, CCR2, CCR3, CCR4, CCR5, CCR6, CCR7, CCR8, CCR9, CEBPB, CRP, CX3CR1, CXCL1, CXCL10, CXCL11, CXCL12, CXCL13, CXCL14, CXCL2, CXCL3, CXCL5, CXCL6, CXCL9, ICEBERG, IFNA2, IL10, IL10RA, IL10RB, IL13, IL13RA1, IL17C, IL1A, IL1B, IL1F10, IL1F5, IL1F6, IL1F7, IL1F8, IL1F9, IL1R1, IL1RN, IL22, IL5, IL5RA, IL8, IL8RA, IL8RB, IL9, IL9R, LTA, LTB, LTB4R, MIF, SCYE1, SPP1, TNFA, CD40LG, TOLLIP, XCR1.

### Protein Interaction Mapping

We built a protein-protein interaction map to model the molecular mechanisms of any biological process induced by proteins encoded by genes significantly increased in response to dental caries.

Physical interactions of proteins encoded by genes on the array were collected using the STRING 8.2 webserver http://string-db.org/[41]. We filtered the resulting information to keep only the largest connected network and include only experimentally verified interactions. A list of the 35 genes corresponding to the unconnected proteins is found in table 2. The resulting network was ported into Cytoscape for further analysis [42]. We then added the TLR4 signal receiver and TGF-beta attenuator we previously described [18], and used the Kyoto encyclopedia of genes and genomes [43] to build downstream signaling pathways, pathway interconnectivity, and aggregate cellular responses.

**Table 2:** The list of 35 unconnected genes excluded from the protein-protein interaction map.

Gene List
C4A, CRP, LTB, LTB4R, CXCL2, CXCL3, CXCL9, CXCL10, CXCL11, CXCL12, CXCL13, CXCL14, CCL15, CCL18, CCL19, CCL20, CCL21, CCL25, XCR1, CX3CR1, CCR6, CCR7, CCR9, IL17C, IL1F5, IL1F6, IL1F7, IL1F8, IL1F9, IL1F10, SPP1, ICEBERG, BCL6, SCYE1, MIF

### Statistical Analyses

The data were analyzed using the analysis of variance followed by the Tukey test for multiple comparisons. Results were considered statistically significant when the P-value was less than 0.05.

## Authors' contributions

All authors have read and agreed with the contents of this manuscript.

Orapin Horst: designed experiments, collected tooth samples and isolated RNA, conducted gene expression analyses, performed some parts of protein interaction mapping, prepared the manuscript.

Jeremy Horst: Performed protein interaction analyses, mapping, and associated bioinformatics analyses and helped with the manuscript preparation.

Ram Samudrala: contributed to protein interaction analyses and manuscript preparation.

Beverly Dale: contributed to gene expression analyses and manuscript preparation.
